# Genome-Wide Identification and Analysis of the *MYC* Gene Family in Cotton: Evolution and Expression Profiles During Normal Growth and Stress Response

**DOI:** 10.3390/genes16010020

**Published:** 2024-12-26

**Authors:** Jingxi Chen, Long Wang, Xiufang Wang, Lu Lu, Peng Han, Caidie Zhang, Min Han, Siyu Xiang, Haibiao Wang, Lizhong Xuan, Zhibo Li, Hairong Lin, Xinhui Nie, Yuanlong Wu

**Affiliations:** 1 Agricultural College, Shihezi University, Shihezi 832003, China; cjx20020222021x@163.com (J.C.); shzuwanglong@163.com (L.W.); lulu520712@163.com (L.L.); han_peng@stu.shzu.edu.cn (P.H.); 18299243164@163.com (C.Z.); 18293220354@163.com (M.H.); 18199123699@163.com (S.X.); lzb_oea@shzu.edu.cn (Z.L.); linhairong@126.com (H.L.); 2 Key Laboratory of Oasis Ecology Agriculture, Xinjiang Production and Construction Crops, Shihezi 832003, China; 3 Xinjiang Production and Construction Corps Seed Management Station, Urumqi 830011, China; wangxiufang9@sina.com; 4 Xinjiang Production and Construction Corps Agricultural Technology Extension Station, Urumqi 830011, China; wanghb0304@163.com (H.W.); xuanlz0991@163.com (L.X.)

**Keywords:** cotton, *MYC* gene family, gene structure, gene expression, evolution

## Abstract

Background: The gene family of myelomatosis (MYC), serving as a transcription factor in the jasmonate (JA) signaling pathway, displays a significant level of conservation across diverse animal and plant species. Cotton is the most widely used plant for fiber production. Nevertheless, there is a paucity of literature reporting on the members of MYCs and how they respond to biotic stresses in cotton. Methods: Bioinformatics analysis was used to mine the MYC gene family in cotton based on InterPro, cottongen, etc. Results: The gene structure, conserved motifs, and upstream open reading frames of 32 *GhMYCs* in *Gossypium hirsutum* were identified. Moreover, it was anticipated that the GT1-motif is the most abundant in *GhMYCs*, indicating that the GT1-motif plays a significant role in light-responsive *GhMYCs*. The expression patterns of *GhMYCs* under biotic stresses including *V. dahliae* and *Aphid gossypii* were evaluated, suggesting that *GhMYCs* in class-1 and -3 GhMYCs, which function as negative regulators, are involved in resistance to verticillium wilt and aphids. The class-3 *GhMYCs* genes were found to be mostly expressed in female tissues. Interestingly, it was also determined that the homeologous expression bias within *GhMYCs* in cotton was uncovered, and results showed that the gene expression of class-1A and class-2 *GhMYCs* in the Dt sub-genome may have a direct impact on gene function. Conclusions: This study provides a research direction for researchers and breeders to enhance cotton traits through manipulating individual or multiple homeologs, which laid a foundation for further study of the molecular characteristics and biological functions of *GhMYC* gene.

## 1. Introduction

The myelomatosis (*MYC*) gene family, which functions as a transcription factor in the jasmonate (JA) signaling pathway, exhibits high conservation across diverse animal and plant species [1,2]. The MYC transcription factor (TF), the Skp–Cullin–F-Box E3 ubiquitin ligase complex SCFCOI1 (COI1, COI1, Coronatine Insensitive 1), and the jasmonate ZIM domain (JAZ) proteins collectively constitute the three core components of the jasmonic acid (JA) signaling pathway [3]. JA is a vital plant hormone that enables plants to withstand mechanical damage, resist infestation by chewing insects, and defend themselves against pathogens that cause vegetative death [4].

The MYC protein family has been demonstrated to be present in numerous plant species, indicating its involvement in a wide range of physiological development processes, including plant growth, development, flower induction, secondary metabolite production, and defense responses [4]. Present studies have revealed the identification of a total of 14 members of the *MYC* gene family in the model plant *Arabidopsis thaliana* [5]. Among them, in the *myc2* mutant, the expression level of *PDF1.2* was found to increase fivefold, with a concomitant enhancement in resistance to *Fusarium necrotrophic wilt* [6], which indicated that the MYC2 transcription factors can regulate the production of specialized defense-related metabolites in response to biological stress. Susceptibility to *Spodoptera littoralis* in the triple mutants of transcription factors AtMYC2, AtMYC3, and AtMYC4 [7] also indicated that AtMYC2 and its homologues AtMYC3 and AtMYC4 play important roles in the defense response [7,8,9,10,11,12,13]. *MYC* is also involved in the defense responses of other plants. The overexpression of *OsMYC2* in response to biological stress has been shown to up-regulate PR genes, thereby conferring resistance to rice bacterial blight [14]. Recent studies have demonstrated that the bHLH transcription factors MTB1 (MYC2-targeted BHLH1), MTB2, and MTB3 are jasmonia-induced and directly activated by MYC2, and the mutants of these genes have been shown to exhibit significant improvement in defense responses without affecting growth and development [15]. Moreover, the double mutants of *zmmyc2a* and *zmmyc2b* in maize exhibited increased susceptibility to insect feeding compared to the wild type [16]. In addition, the MYC protein has been demonstrated to co-regulate the aromatic amino acid defense mechanism with JAZ, thereby increasing resistance to necrotic pathogens while concomitantly reducing resistance to phytophagous insects [17].

Meanwhile, MYC protein family members not only play a crucial role in biological stress but also serve an indispensable role in abiotic stress. Atmyc2 mutants show higher rates of seed germination and root growth under high-salinity conditions [10]. The drought tolerance of AtMYC2-overexpressing transgenic *Arabidopsis* plants was markedly enhanced by the simultaneous activation of MYC2 and inhibition of ATAF1 [8,12]. In contrast, *med25* mutations that physically interact with MYC2 demonstrate high sensitivity to salt and significantly reduced seed germination rates under salt treatment compared to the wild type [18]. Phenotypic comparison of *Arabidopsis myc2* and *med25* mutants has demonstrated that MYC2 exhibits a negative regulatory effect on salt tolerance via *MED25*. The atmyc2/3/4 mutation mitigates the inhibition of root cell elongation caused by salt [19]. Studies have also shown that MYC2 can inhibit SlRR26 (a B-type response regulator of the cytokinin pathway), which is a pivotal regulator of stomatal movements in response to JA under conditions of drought stress [20]. MYC2 enhances salt tolerance in rice by regulating the expression of the cyclophilin chaperone OsCYP2 [21]. Moreover, MYC2, which plays a pivotal role in plant low-temperature stress, activates the transcriptional cascade regulation of ICE-CBF/DREB-COR by its interaction with the INDUCER OF CBF EXPRESSION (ICE), subsequently binding to CBFs (C-REPEAT BINDING FACTORS)/DREB (dehydration response element binding factors) promoters and up-regulating downstream gene expression [22]. In addition, MYC2 has been identified to interact with INDUCER OF CBF EXPRESSION 1 (ICE1) in MeJA-induced cold resistance in wheat and bananas [23,24]. MdMYC2 is capable of enhancing cold tolerance in apples through its ability to bind to the G-box element located within the MdCBF1 promoter [25]. Finally, Under LD and SD conditions, *myc2/3* and *myc2/4* double mutants and *myc2/3/4* triple mutants displayed consistent early flowering phenotypes, showing that *MYC* gene family plays an essential role in growth and development by interaction with jasmonate [26].

Cotton is a globally significant cash crop, a unique commodity, and a crucial strategic material for national economic advancement. However, there have been a limited number of reports indicating that *MYCs* may be involved in the cotton defense response, particularly at the genome-wide level. Here, the genome-wide annotation and members of *MYC* genes in the *G. hirsutum* are investigated, and we report their phylogenetic relationships, structural and conserved motifs, cis-acting elements and transcription factors, tissue expression patterns, the varying bias in homeolog expression, and their response to *V. dahliae* and *A. gossypii*. This research establishes a foundation for further research on the functional roles of the *GhMYCs* gene family.

## 2. Results

### 2.1. Genome-Wide Identification of MYC Gene Family in Upland Cotton

To identify myelocytomatosis protein (MYC) sequences in upland cotton, a total of thirty-two predicted GhMYC protein sequences were identified using InterPro and cottongen and confirmed using NCBI Batch CD-Search (Tables S1 and S2). Bioinformatics analysis showed that these sequences have protein lengths of 421–684 aa (Table 1). The largest MYC (*Ghir_A08G019450.1*) consists of 684 aa, whereas the smallest MYC (*Ghir_A08G013170.1*) comprises only 421 aa (Table 1). The molecular weights of these amino acid sequences span a range, varying between 47.05 and 76.48 kDa. All the GhMYCs proteins were predicted to be acidic, with PI ranging from 4.97 to 6.81 (Table 1). Subcellular localization prediction indicated that GhMYC proteins predominantly reside within the nucleus. However, it is noteworthy that the specific protein *Ghir_A08G011490.1* exhibited dual localization, being found not only in the nucleus but also within the chloroplasts (Table 1). The predicted instability index, aliphatic index, and grand average of hydropathicity ranged from 39.66 to 70.55, 67.79 to 89.75, and –0.723 to –0.348, respectively (Table 1), indicating that GhMYCs encoded unstable hydrophilic proteins. These findings suggest that the characteristics of instability, hydrophilicity, and nuclear localization are commonly associated with MYC proteins.

### 2.2. Phylogenetic Analysis of the MYC Gene Family

**Figure 1:**
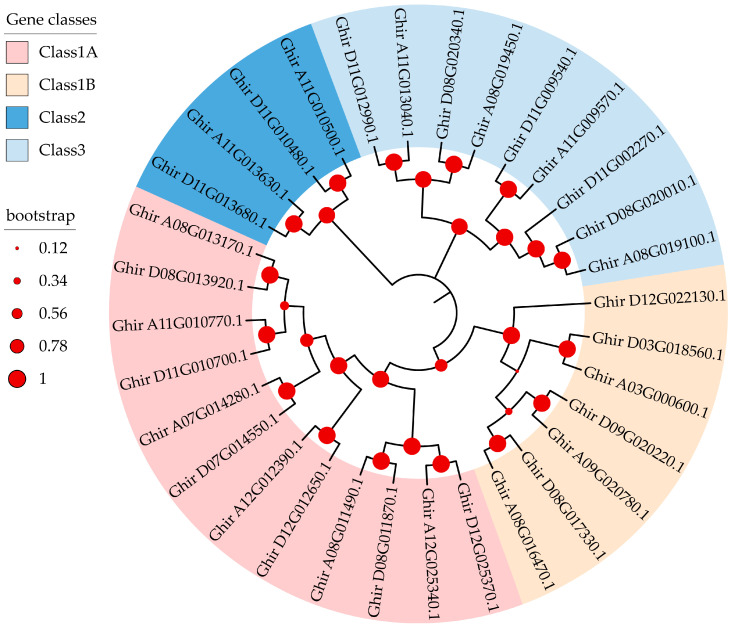
Phylogenetic tree of MYC proteins in upland cotton. The phylogenetic tree was constructed using the MEGA X with 1000 bootstrap replicates. Based on the phylogenetic tree, the *MYC* gene family was divided into three classes, which are shown in different colors. Among them, class 1 was divided into two subgroups. The bootstrap value is shown as a red ring.

To uncover the phylogenetic relationships among the 32 *G. hirsutum GhMYCs*, the phylogenetic tree was reconstructed using an ML procedure. It showed that *GhMYCs* are clustered into four groups. There are 12 members in class 1A; 7 members in class 1B; 4 members in class 2; and 9 members in class 3 (Figure 1). Among them, class 1 contains the most members and can be divided into two subgroups, suggesting that class-1 *GhMYCs* have undergone duplication evolutionary events and gene function divergence. A pairwise comparison of the 32 full-length *GhMYC* sequences revealed notable features (Figure S1). All class-1 (including 1A and 1B) *GhMYCs* showed > 35%. The pairwise sequence identity for all class-2 and class-3 *GhMYCs* was found to be greater than 24%. The box plot revealed that the protein sequence identities of class-1B *GhMYCs* were significantly higher than those of other classes, except for class-2 *MYCs* (independent-sample *t*-test, *p* < 0.0001) (Figure S1). This suggests that the degree of sequence divergence among class-1A/class-3 *MYCs* was greater than that among class-1B *GhMYCs*. It is noteworthy that the protein sequence identities among the class-2 *GhMYCs* had undergone a substantial alteration, indicating that the degree of sequence divergence among the class-2 *GhMYCs* was high (Figure S1).

### 2.3. Analysis of the Structural and Conserved Motifs of GhMYCs

**Figure 2:**
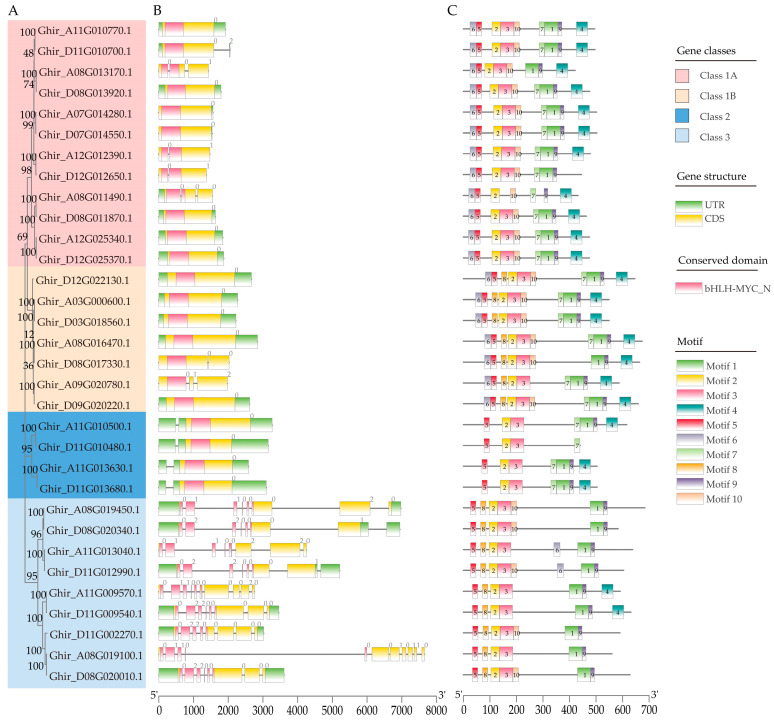
A detailed examination of the gene structure and architecture features of the conserved protein motifs in *GhMYC* genes. (**A**) The phylogenetic tree was constructed using the full-length sequences of the GhMYC proteins. (**B**) The exon–intron structure of GhMYCs is presented herewith. The untranslated regions, exons, and introns are represented by light green boxes, light yellow boxes, and horizontal lines, respectively. The red boxes represent the bHLH-MYC_N domain. (**C**) Ten types of conserved motifs, indicated by different colored boxes, were predicted in the GhMYCs protein sequences. The primary distinction between class 1A and class 1B is the absence or presence of motif 8.

To gain further insight into the evolutionary history of the *GhMYC* genes, an investigation was conducted into the exon–intron organization, functional domains and conserved motifs of the *GhMYCs*. The results of this analysis demonstrated that all *GhMYCs* contain the bHLH-MYC_N domain and that the number of exons present is from zero to ten (Figure 2A,B). Interestingly, the number of exons in class-3 GhMYCs was more than that in other GhMYCs, indicating that there is functional divergence between class-3 GhMYCs and other GhMYCs (Figure 2B). An online MEME analysis was conducted to reveal ten conserved motifs present across the 32 *GhMYCs* genes (Table S3). There are 10 motifs in class 1B, with *Ghir_D08G017330.1* being an exception, featuring only 9 motifs (Figure 2C), while other classes of GhMYCs exhibit 4–9 motifs (Figure 2C). Interestingly, motifs 2 and 5 are distributed in all GhMYCs (Figure 2C). Further analysis revealed that the amino acid with the highest frequency in motif 2 and motif 5 is F (phenylalanine) and W (tryptophan), respectively (Figure S3), which suggests that these amino acids may play vital roles in the function of GhMYCs. Further, the multiple-sequence alignment of all MYC proteins from *G. hirsutum*, *G. barbadense*, *G. raimondii*, *A. thaliana*, *Oryza sativa*, *Selaginella mollendorffii*, *Nymphaea colorata*, *Amborella trichopoda*, and *Physcomitrella patens* showed that hydrophobic core amino acid residues (VALFIs) were observed in almost all MYC protein sequences, indicating that these residues are crucial for the folding and fundamental functions of MYC proteins (Figure S4).

Present studies showed the variations in upstream open reading frame (uORF) shape phenotypic diversity in plants [27]. However, the uORF functions of *GhMYCs* were still unclear. Based on the method as described by Zhang et al. [28], 18 *MYCs* were identified in *Gossypium raimondii*, and the uORF was detected in 12 out of 18 *MYC* genes in *G. raimondii*. Then, the homologous relationship with the *MYC* gene between *G. raimondii* and *G. hirsutum* was determined, and it showed that there is the most uORF in *Ghir_A11G009570.1*/*Ghir_D11G009540.1*. Interestingly, uORFs were not detected in class-1A *GhMYCs*, suggesting that class-1A *GhMYCs* may be functionally divergent by losing the uORF throughout their evolution (Table S4). However, the impact on upland cotton has yet to be explored in future studies.

### 2.4. Prediction of Cis-Acting Elements and Transcription Factors Among the GhMYCs

**Figure 3:**
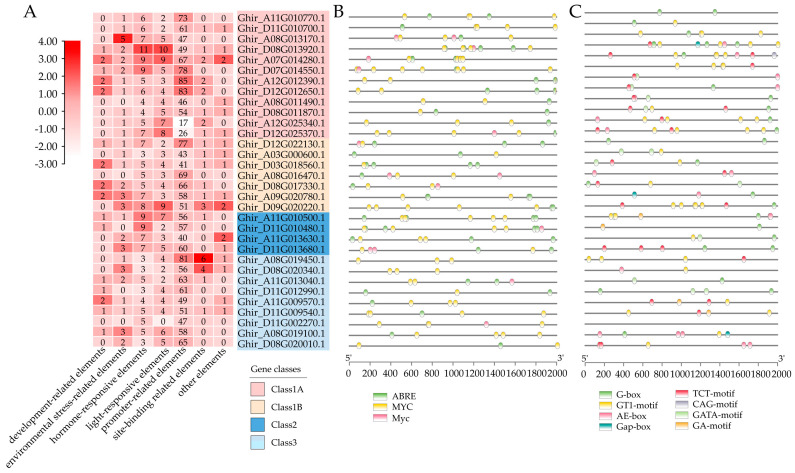
In the 2000 bp promoter regions of the *GhMYCs*, cis-acting elements were predicted. (**A**) A schematic representation of the number of cis-acting elements detected in the promoter region of each *GhMYC* gene. All cis-acting elements were classified into seven distinct categories, with the number of elements within each category normalized by column. (**B**,**C**) The distribution of hormone-responsive (**B**) and light-responsive (**C**) elements in the promoter region of the *GhMYC* gene is examined.

In order to predict the cis-acting elements among the *GhMYCs*, the 2000 bp upstream promoter sequence of *GhMYCs* genes were collected by using TM-1 genome data [29] and analyzed by using PlantCARE. This revealed that a total of 2286 cis-acting elements, representing 27 types, were predicted, and they were divided into seven groups including development-related, environmental stress-related, hormonal response, promoter-related, site binding, and other functional elements (Table S5). Among them, the promoter-related elements group, with 17-85 elements, was the group with the largest number of elements (Figure 3A). There were more elements in class 1A and class 2 than in class 1B or class 3 (Figure 3A). The prediction of 187 hormone-related components was divided into three categories (ABRE, MYC and Myc), the majority of which were related to ABA and JA (Figure 3B), which indicated that *GhMYC*s might have the potential for self-regulation. There were 140 elements related to light-responsiveness that were predicted in eight categories, including G-box, GT1 motif, AE-box, Gap-box, TCT motif, CAG motif, GATA motif, and GA motif (Figure 3C). Among them, the GT1 motif (47 out of 140) is the most abundant, indicating that the GT1 motif plays an important role in light-responsive *GhMYCs*.

**Figure 4:**
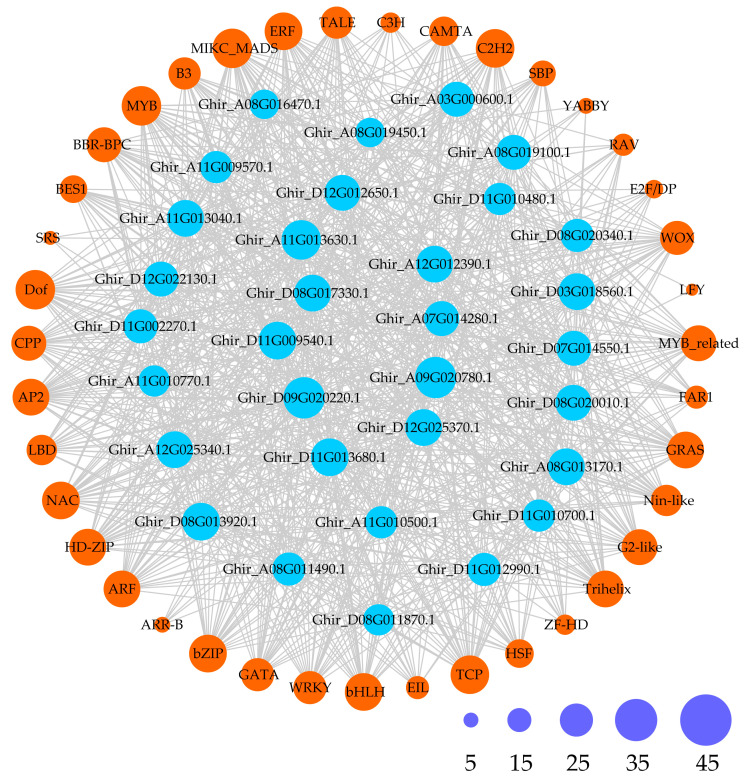
Regulatory network between *GhMYCs* and potential transcription factors (TFs) was demonstrated. Blue rings with gene ID represent *GhMYCs*, orange rings with TF name represent possible TFs, and gray lines represent potential regulatory relationships. Ring size represents degree of potential regulatory relationships between *GhMYCs* and TFs. Size and number of interactions are shown by purple rings.

Furthermore, to provide a comprehensive demonstration of the regulatory network of GhMYCs in upland cotton, the potential TFs of GhMYCs were predicted, which revealed that a total 39 types of transcription factors were predicted to be involved in regulating *GhMYC* expression (Figure 4 and Table S6). These different *GhMYC* genes were regulated by a range from 21 to 34 transcription factors. In detail, the number of TFs in each class is as follows: class 1A—23 to 34; class 1B—24 to 30; class 2—25 to 29; class 3—21 to 34 (Table S7). Only the MIKC_MADS, MYB, and Dof transcription factors were conserved in the regulation of all *GhMYC* expression, indicating that the MIKC_MADS, MYB, and Dof transcription factors play important roles in regulating *GhMYC* expression (Table S7). Moreover, the specific transcription factor LFY was only present in *Ghir_D07G014550* and *Ghir_A08G019450*. These results indicated that class-3 *GhMYCs* underwent more intense functional differentiation during evolution.

### 2.5. Tissue Expression Patterns of GhMYCs

**Figure 5:**
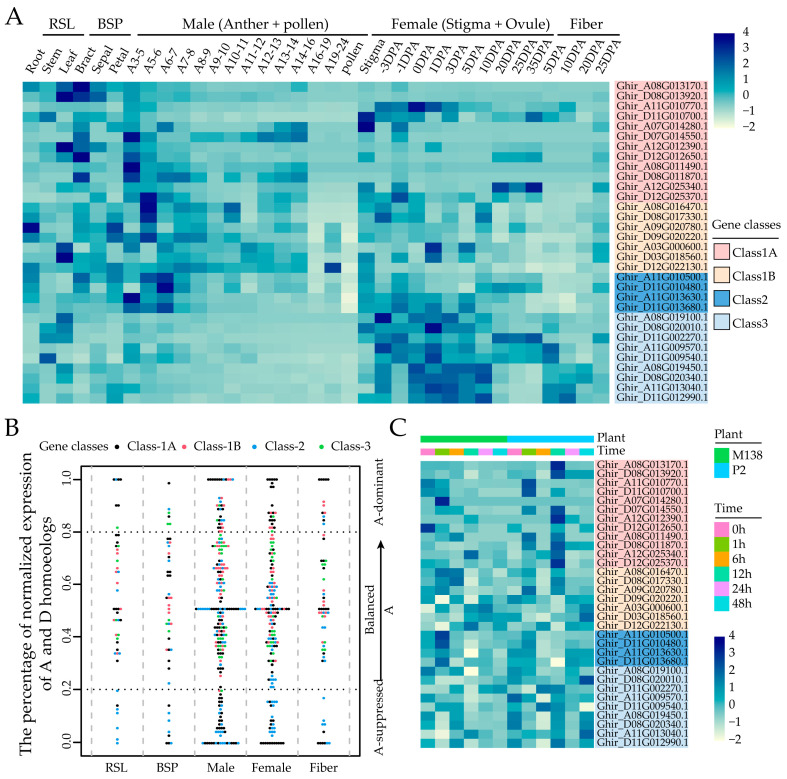
The expression pattern of *GhMYCs*. (**A**) A heatmap was constructed to demonstrate the tissue-specific expression of *GhMYCs*. RSL: root, stem, and leaf; BSP: bract, sepal, and petal; Anther: different anther development stages are based on bud length—for example, A3–5 represents anther in 3–5 mm bud; Ovule: ovule at different developmental stages; Fiber: Fiber at different developmental stages; DPA: days post anthesis. (**B**) The bias in homologous gene expression was observed to vary between tissues within *GhMYCs* genes in the At sub-genome. (**C**) A heatmap was constructed to demonstrate the expression pattern of *GhMYCs* between *V. dahliae*-resistant and -susceptible cotton cultivars after infection with *V. dahliae isolates*, strong pathogenicity strain V991.

To gain a comprehensive understanding of the functions of *GhMYCs*, the spatial and temporal expression analysis of all *GhMYCs* was determined using RNA-seq data. This analysis was performed in each of the tissues, including RSL (root, stem, and leaf), BSP (bract, sepal, petal), male (different-stage anthers and pollen), female (stigma and ovule), and fiber (Figure 5A and Table S8). These findings revealed that that *GhMYC* genes are predominantly expressed in vegetative and female tissues, exhibiting minimal expression in male tissues, particularly in pollen (Figure 5A). Moreover, class-3 *GhMYCs* genes were found to be predominantly expressed in female tissues, which suggests that class-3 *GhMYCs* play an important role in female development (Figure 5A).

It is noteworthy that the bias in homeologous expression exhibits variability among tissues within *GhMYCs*, as observed in tetraploid upland cotton [28,30]. To determine patterns of homeologous expression within *GhMYCs*, the distribution of *GhMYC* genes across *G. hirsutum* chromosomes was investigated, and it was revealed that there is an uneven localization of *GhMYCs* across the 12 *G. hirsutum* chromosomes (Figure S5). Then, the gene pairs were identified through collinearity analysis, revealing that a total of 15 gene pairs within *GhMYCs* were detected, except for *Ghir_D12G022130* (Class-1B) and *Ghir_D11G002270* (Class-3). These were distributed as follows: six, three, two, and four pairs in class 1A, class 1B, class 2 and class 3, respectively (Figure S5). The bias in the homeologous expression of *GhMYCs* was analyzed. If the same genes have similar transcript abundance in both sub-genomes, the balanced expression term applies. If there are more or fewer transcripts in one sub-genome, they are A-suppressed or A-dominant. The results demonstrated that most A-suppressed genes were concentrated in class 1A and class 2, whereas the A-dominant genes were distributed across all classes (Figure 5B), which suggests that the gene expression of class-1A and class-2 *GhMYCs* in the Dt sub-genome may have a direct impact on gene function. Further analysis showed that *GhMYC* genes were mainly balanced in vegetative and reproductive tissue (Figure 5B), which suggests that the divergence in gene function between the At and Dt sub-genomes is not readily apparent. To gain further insight into the evolutionary process, the Ka/Ks ratios of 15 gene pairs within *GhMYCs* were calculated as a measure of selection pressure. The results demonstrated that the Ka/Ks ratios of the *GhMYCs* homeologous pairs exhibited a range from 0.0825 to 1.0902 (Table S9). Among them, only the pair *Ghir_A12G012390* and *Ghir_D12G012650* demonstrated positive selection with a Ka/Ks ratio greater than 1. The other *GhMYC* homeologous pairs were subjected to significant purification during the evolutionary process, with the pair *Ghir_A12G025340* and *Ghir_D12G0225370* undergoing the most pronounced changes. Interestingly, these two pairs were classified as class 1A, indicating that a substantial evolutionary shift has occurred within class 1A.

### 2.6. Expression of GhMYCs Under Biotic Stress

To explore whether *GhMYCs* are involved in the response to *V. dahliae* and *A*. *gossypii*, the expression patterns of *GhMYCs* in cotton plants infiltrated with V. dahliae (V991) and *A. gossypii* were examined, revealing that the gene expression of *GhMYC* genes, expect for class 2, exhibited a higher expression in the *V. dahliae*-susceptible cotton cultivar (P2) than in the *V. dahliae*-resistant cotton cultivar (M138) 12 h after infection with V991 (Figure 5C and Table S10), which suggests that the GhMYCs in class 1 and class 3 played a role in the resistance of cotton to verticillium wilt as a negative regulator. The expression pattern analysis of *GhMYCs* after infection with *A. gossypii* showed that only class-1A genes (*Ghir_A07G014280* and *Ghir_D08G011870*) and class-3 (*Ghir_A08G019100*) *GhMYC* genes were observed to be more highly expressed in the aphid-susceptible *G. hirsutum cultivar* (Z50) than in the aphid-resistant *G. hirsutum cultivar* (Z61), revealing that the *GhMYCs* in class 1 and class 3 played a role in the response to *A. gossypii* as a negative regulator (Table S11). These results showed that the *GhMYCs* in classes 1 and 3 (particularly *Ghir_A07G014280*, *Ghir_D08G011870* and *Ghir_A08G019100*) function as negative regulators, playing a role in resistance to verticillium wilt and aphids. However, further experiments are required to confirm these findings.

## 3. Discussion

### 3.1. The Conserved Motif and Sequence Characterization of GhMYCs

The MYC protein family plays a key role in a multitude of physiological development processes, including plant growth, development, flower induction, secondary metabolite production, and defense responses [4,8]. Consequently, they represent promising targets for crop breeding and improvement. However, relatively few studies have documented the function of the *MYC* gene family. In this study, a total of thirty-two predicted *GhMYCs* genes were identified and assigned to three conserved subfamilies (Figure 1). Further, the structural domains of GhMYCs were found to be conserved, displaying characteristics of instability and hydrophilicity and exhibiting nuclear localization (Figure 2). These features are likely to be typical of MYC proteins, as evidenced by the phylogenetic tree, the gene structure, and the analysis of the conserved motifs. These results suggested that GhMYCs have evolutionarily conserved functions during evolution and may play a crucial role in a multitude of physiological development processes. Class-1B GhMYCs are homologous to MYC2, which has been reported most recently [10,21,31]. Interestingly, the pairwise sequence identity of class 1B, with the most abundant motifs, was highest, which indicated that class-1B MYCs have evolutionarily conserved functions and may play a central role in defense responses. On the contrary, the results of the number of exons and regulated transcription factors in class 3 showed that there is a functional divergence between class-3 GhMYCs and other GhMYCs (Figure 2B), and uORFs were not detected in class-1A GhMYCs, suggesting that class-1A GhMYCs may be functionally divergent by losing the uORF throughout their evolution (Table S4). This structural basis underpins the functional diversity of the *MYC* gene family. Furthermore, the GT1 motif is the most abundant, indicating that the GT1 motif of *GhMYCs* plays a pivotal role in light-responsive processes, which indicates that *GhMYCs* may play an important role in light-responsive mechanisms. However, more experiments are needed in the future.

### 3.2. Function Analysis of GhMYC Genes Based on Gene Expression Pattern

Cotton is one of the world’s most significant cash crops, a unique commodity, and a crucial strategic material for national economic advancement [32]. However, it is often subjected to a multitude of biotic stresses, including Verticillium wilt, aphids, cotton bollworm, red spider, and so on [33,34]. Present studies showed that *MYCs* play an important role in the defense responses of plants to biotic stresses [4,14,17]. Resistance to *F. necrotrophic wilt* was enhanced in *myc2* mutants [6]. Moreover, it has been shown that the increased expression of *OsMYC2* in response to biological stress leads to the up-regulation of PR genes, thereby imparting resistance to rice against bacterial blight [14]. In this study, the expression pattern analysis showed that *GhMYCs* in classes 1 and 3 (particularly *Ghir_A07G014280*, *Ghir_D08G011870,* and *Ghir_A08G019100*), which function as negative regulators, play a role in resistance to verticillium wilt and aphids (Figure 5). These results provide genetic resources for cotton resistance breeding to verticillium wilt and aphids.

Moreover, the bias analysis of the homeologous expression in *GhMYCs* showed that the class-3 *GhMYCs* genes were found to be predominantly expressed in female tissues, which suggests that class-3 *GhMYCs* play an important role in female development (Figure 5A), and the gene expression of class-1A and class-2 *GhMYCs* in the Dt sub-genome may have a direct impact on gene function. As described by previous studies, gene expression and domestication bias existed in the cotton subgroups At and Dt [30,35]. However, more experiments are needed to confirm the function, gene expression, and domestication bias of *GhMYCs* in the future.

## 4. Conclusions

In this study, the gene structure, conserved motifs, and uORFs of *GhMYCs* in upland cotton were identified. Moreover, the expression patterns of *GhMYCs* under biotic stresses, including V. dahliae and *A*. *gossypii*, and the homeologous expression bias within *GhMYCs* were evaluated. There results provide a research direction for researchers and breeders to enhance cotton traits through manipulating individual or multiple homeologs, which lays a foundation for further study of the molecular characteristics and biological functions of the *GhMYC* gene.

## 5. Materials and Methods

### 5.1. Identification of the MYC Gene Family

To identify the *MYC* gene family in *G. hirsutum*, MYC protein sequences of *A. thaliana* (AT1G01260) were used as queries in InterPro (http://www.ebi.ac.uk/interpro/). Then, the candidate genes in *G. hirsutum* were used as queries in a BLAST search (score > 50, E-value < 0.01) in cottongen (https://www.cottongen.org/, accessed on 25 February 2024) with the *G. hirsutum* TM-1 genome [29]. Then, the gene sequence and location information were also derived from the TM-1 genome [29]. The NCBI’s Batch CD-Search function (https://www.ncbi.nlm.nih.gov/Structure/bwrpsb/bwrpsb.cgi, accessed on 27 February 2024) was employed to corroborate the presence of the characteristic bHLH-MYC_N domain (NCBI, cd13983) in the candidate *MYC* genes. Among them, the genes with an incomplete bHLH-MYC_N domain were eliminated. The characteristics of the *MYC* gene candidates in *G. hirsutum*, including their protein length, isoelectric point (pI), and molecular weight (MW), were calculated using ExPASy tools, facilitated by TBtools. Additionally, the subcellular localization of these proteins was forecasted using the Plant-mPLoc algorithm.

### 5.2. Phylogenetic Analysis and Molecular Evolution Analyses

The sequences of the *MYC* kinase gene family in *G. hirsutum* were sourced from cottongen. A comprehensive alignment of the complete protein sequences of the *GhMYC* gene family was conducted using Clustal-X version 1.8 [36]. Subsequently, a phylogenetic tree was constructed using the maximum likelihood (ML) method, with the following options enabled: the Jones–Taylor–Thornton (JTT) amino acid substitution model, γ-distributed rates among sites, and 1000 bootstrap replicates in MEGA-X (https://www.megasoftware.net/, accessed on 17 April 2024). Finally, the tree was further annotated and beautified by using iTOL (https://itol.embl.de/, accessed on 16 May 2024) [37]. Moreover, the *MYC* gene family derived from *G. barbadense*, *G. raimondii*, *A. thaliana*, *O. sativa*, *S. moellendorffii*, *N. colorata*, *A. trichopoda,* and *P. patens* was identified from Ensembl Plants (https://plants.ensembl.org/info/data/ftp/index.html, accessed on 21 June 2024) [38], and multiple-sequence alignment was performed as described above. The rates of synonymous (Ks) and nonsynonymous (Ka) substitutions for pairwise comparisons within the *GhMYC* gene family were determined using the PAML package [39,40].

### 5.3. Multiple Sequence Alignment of MYC Proteins

The protein sequences of members of the MYC family were employed to generate a multiple-sequence alignment and to conduct visualization analyses using Clustal-X (http://www.clustal.org/, accessed on 28 June 2024) and Jalview, respectively.

### 5.4. Analysis of Conserved Motifs, Gene Structure, Functional Domains, and 3D Structure

The conserved motifs within the *GhMYCs* gene family were identified using the MEME program [41], adhering to its default settings and specifying a maximum of 10 motifs to be detected. The GFF3 data of the GhMYCs proteins and the reference genome of *G. hirsutum* [29] were downloaded from cottongen. Then, the gene structure and functional domains were subjected to analysis and visualization utilizing the NCBI Batch CD-Search [42] and TBtools. In order to acquire the three-dimensional structure of GhMYC proteins, the GhMYC protein sequences were submitted to SWISS-MODEL (https://swissmodel.expasy.org/) with default algorithm parameters.

The uORFs of MYCs were detected using uORFlight (http://www.rnairport.com:443/Tool_uORFFinder.php, accessed on 17 August 2024) [43]. The uORFs of GhMYCs were detected using sequences for ICCu (initiation codon context for upstream open reading frames) and ICCm (initiation codon context for major open reading frames).

### 5.5. Promoter Cis-Acting Elements and TF Prediction

To identify the cis-acting elements that act as promoters for the *GhMYCs* genes, the promoter sequence of the genes, which had a length of 2000 bp, was obtained. Following this, the sequence was predicted by PlantCARE [44] and visualized by TBtools. Additionally, the transcription factors associated with *GhMYC* genes were forecasted using PlantRegMap, focusing on *A. thaliana* as the reference species. Cytoscape 3.6.0 software was employed for the visualization of the target relationships between transcription factors and *GhMYCs* [45].

### 5.6. Gene Expression Analysis

The tissue expression data of *GhMYCs* were downloaded from Zhang et al. (2022) [46] and visualized using R (https://www.r-project.org/). Subsequently, the homologous gene expression bias of *GhMYCs* was determined in accordance with the methodology described by Ramírez-González et al. (2018) [47]. Furthermore, the expression patterns of infiltration with *V. dahliae* (V991) and *A. gossypii* were obtained from the literature [34,48]. The cultivars M138 and P2 were derived from a MAGIC population and were selected as representatives of *V. dahliae*-resistant and -susceptible cotton, respectively. Xinluzao 61 and Xinluzao 50 were selected as representatives of aphid-resistant and -susceptible *G. hirsutum*, respectively.

## Figures and Tables

**Table 1:** Detailed information of the 32 predicted GhMYC proteins in upland cotton.

Sequence ID	Gene Location	Strand	CDS	Protein Length (aa)	Amino Acids MW (kDa)	Theoretical pI	Predicted Localization	Instability Index	Aliphatic Index	Grand Average of Hydropathicity
Ghir_A03G000600.1	Ghir_A03:980039-982310	+	1650	549	60.99	5.82	Nucleus.	48.55	76.36	−0.60
Ghir_A07G014280.1	Ghir_A07:29260134-29261695	-	1509	502	56.09	5.21	Nucleus.	55.4	78.03	−0.45
Ghir_A08G011490.1	Ghir_A08:78260647-78262193	+	1299	432	48.21	6.15	Chloroplast. Nucleus.	44.73	81.2	−0.35
Ghir_A08G013170.1	Ghir_A08:93976363-93977793	+	1266	421	47.05	6.22	Nucleus.	43.14	84.77	−0.40
Ghir_A08G016470.1	Ghir_A08:107604743-107607585	+	2025	674	73.57	5.46	Nucleus.	54.11	70.49	−0.58
Ghir_A08G019100.1	Ghir_A08:113779847-113787499	+	1683	560	62.40	5.78	Nucleus.	52.97	88.96	−0.41
Ghir_A08G019450.1	Ghir_A08:114199928-114206901	+	2055	684	76.48	5.05	Nucleus.	66.57	71.9	−0.66
Ghir_A09G020780.1	Ghir_A09:77127717-77129696	-	1764	587	63.77	5.52	Nucleus.	50.77	73.8	−0.56
Ghir_A11G009570.1	Ghir_A11:8503876-8506632	+	1776	591	66.83	5.37	Nucleus.	49.02	89.75	−0.38
Ghir_A11G010500.1	Ghir_A11:9603951-9607216	-	1851	616	68.18	6.19	Nucleus.	47.89	74.09	−0.54
Ghir_A11G010770.1	Ghir_A11:10151540-10153460	+	1488	495	55.19	5.37	Nucleus.	55.23	86.67	−0.42
Ghir_A11G013040.1	Ghir_A11:13511016-13515266	+	1917	638	71.78	5.83	Nucleus.	60.7	80.13	−0.54
Ghir_A11G013630.1	Ghir_A11:14785560-14788144	+	1515	504	55.69	5.51	Nucleus.	45.38	76.59	−0.47
Ghir_A12G012390.1	Ghir_A12:80898036-80899508	+	1440	479	53.46	5.19	Nucleus.	43.44	82.23	−0.51
Ghir_A12G025340.1	Ghir_A12:104279639-104281482	+	1428	475	53.05	6.35	Nucleus.	43.55	78.13	−0.48
Ghir_D03G018560.1	Ghir_D03:52297122-52299344	-	1650	549	60.70	5.96	Nucleus.	50.25	76.74	−0.60
Ghir_D07G014550.1	Ghir_D07:22069145-22070681	-	1512	503	56.22	5.2	Nucleus.	54.13	78.67	−0.47
Ghir_D08G011870.1	Ghir_D08:40245698-40247330	-	1392	463	52.00	6.24	Nucleus.	49.07	81.23	−0.36
Ghir_D08G013920.1	Ghir_D08:47888044-47889836	-	1431	476	53.07	6.36	Nucleus.	42.21	79.5	−0.50
Ghir_D08G017330.1	Ghir_D08:55836510-55838535	+	1995	664	72.32	5.29	Nucleus.	51.17	71.7	−0.54
Ghir_D08G020010.1	Ghir_D08:60585556-60589165	+	1887	628	70.24	5.95	Nucleus.	53.7	86.61	−0.47
Ghir_D08G020340.1	Ghir_D08:61033485-61040431	+	1752	583	65.29	4.97	Nucleus.	70.55	67.79	−0.72
Ghir_D09G020220.1	Ghir_D09:48417384-48420001	-	1980	659	71.78	5.21	Nucleus.	49.37	72.23	−0.60
Ghir_D11G002270.1	Ghir_D11:1856954-1859976	+	1773	590	65.88	5.73	Nucleus.	57.77	81.59	−0.52
Ghir_D11G009540.1	Ghir_D11:7909491-7912950	+	1896	631	71.21	5.48	Nucleus.	49.39	88.24	−0.42
Ghir_D11G010480.1	Ghir_D11:8889334-8892486	-	1326	441	48.81	6.81	Nucleus.	44.54	69.21	−0.59
Ghir_D11G010700.1	Ghir_D11:9307960-9310014	+	1491	496	55.45	5.35	Nucleus.	52.29	83.53	−0.45
Ghir_D11G012990.1	Ghir_D11:12048161-12053376	+	1815	604	68.51	5.25	Nucleus.	68.22	78.21	−0.63
Ghir_D11G013680.1	Ghir_D11:12872213-12875310	+	1515	504	55.57	5.59	Nucleus.	44.6	78.73	−0.44
Ghir_D12G012650.1	Ghir_D12:41821932-41823302	+	1338	445	49.55	5.32	Nucleus.	39.66	80.18	−0.47
Ghir_D12G022130.1	Ghir_D12:56236545-56239215	+	1941	646	71.00	5.47	Nucleus.	50.61	71.56	−0.58
Ghir_D12G025370.1	Ghir_D12:59331943-59333814	+	1428	475	53.04	6.4	Nucleus.	44.57	78.13	−0.48

## Data Availability

All data generated or analyzed during this study are included in this published article and its additional files. The datasets used and analyzed during the current study are available from the corresponding author on reasonable request.

## Supplementary Materials

The following supporting information can be downloaded at: https://www.mdpi.com/article/10.3390/genes16010020/s1, Figure S1. Pairwise sequence identity of full-length MYC proteins. A, B, C, and D represent pairwise sequence identities of class-1A, class-1B, class-2, and class-3 MYC proteins, respectively. AB, AC, AD, BC, BD, and CD represent pairwise sequence identities between class-1A and -1B MYC proteins, class-1A and -2 MYC proteins, class-1A and -3 PG proteins, class-1B and -2 MYC proteins, class-1B and -3 MYC proteins, and class-2 and -3 MYC proteins. The box plot shows the median (black line) and interquartile range (box). Outliers are shown as circles outside of the whiskers. Figure S2. The 3D structure of MYC proteins. The 3D structure of class-1A (A), class-1B (B), class-2 (C), and class-3 (D) in the At sub-genome and part of the Dt sub-genome are shown. Figure S3. The sequence of motifs in GhMYCs. A total of 10 conserved motifs were identified in GhMYCs, and the sequence of motifs are shown. Figure S4. The conserved sequence of MYC proteins from the nine plant species. A total of 147 MYC proteins from G. hirsutum, *G. barbadense*, *G. raimondii*, *A. thaliana*, *O. sativa*, *S. mollendorffii*, *N. colorata*, *A. trichopoda*, and *P. patens* were used to perform multiple sequence alignment and visualization by using Clustal-x, and Jalview. The amino acid residues in the MYCs are colored. Red arrows indicate highly conserved positions of VALFI (hydrophobic core amino acid residues) residues. Figure S5. Genomic localization and collinearity analysis of *GhMYCs***.** The genomic localization and collinearity analysis of *GhMYCs* are shown in the At sub-genome and part of the Dt sub-genome of the cotton genome by using PAML.
