# Supplementary material for: Genome-Wide Identification and Analysis of the MYC Gene Family in Cotton: Evolution and Expression Profiles During Normal Growth and Stress Response
Source: Genes (Basel). 2024 Dec 26;16(1):20. doi: 10.3390/genes16010020 (PMC11765489; doi:10.3390/genes16010020)
Supplement: Supplementary file 1 [file genes-16-00020-s001.zip › genes-3377153-supplementary.pptx]

## Slide 1
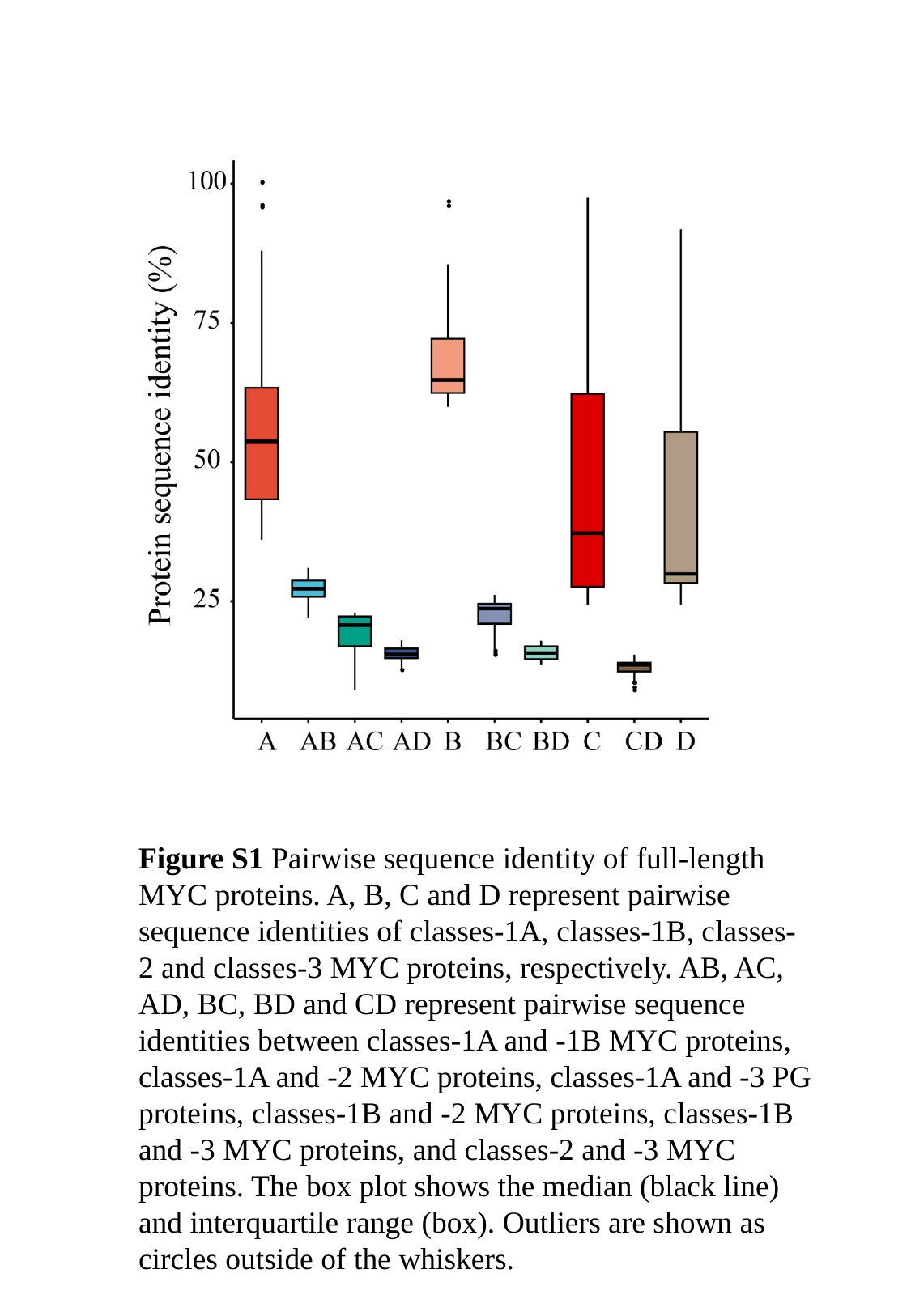

Figure S1 Pairwise sequence identity of full-length MYC proteins. A, B, C and D represent pairwise sequence identities of classes-1A, classes-1B, classes-2 and classes-3 MYC proteins, respectively. AB, AC, AD, BC, BD and CD represent pairwise sequence identities between classes-1A and -1B MYC proteins, classes-1A and -2 MYC proteins, classes-1A and -3 PG proteins, classes-1B and -2 MYC proteins, classes-1B and -3 MYC proteins, and classes-2 and -3 MYC proteins. The box plot shows the median (black line) and interquartile range (box). Outliers are shown as circles outside of the whiskers.

## Slide 2
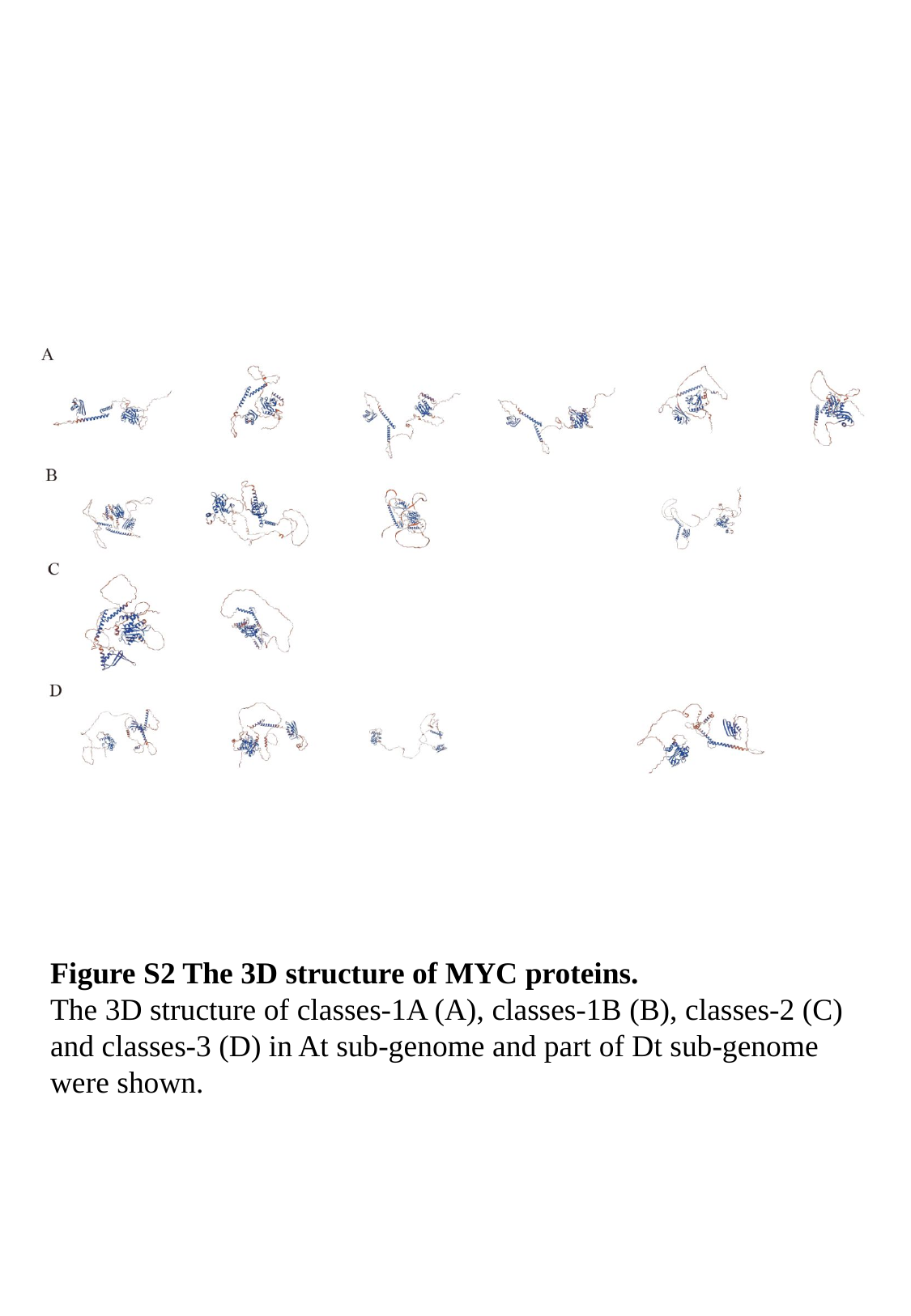

Figure S2 The 3D structure of MYC proteins.
The 3D structure of classes-1A (A), classes-1B (B), classes-2 (C) and classes-3 (D) in At sub-genome and part of Dt sub-genome were shown.

## Slide 3
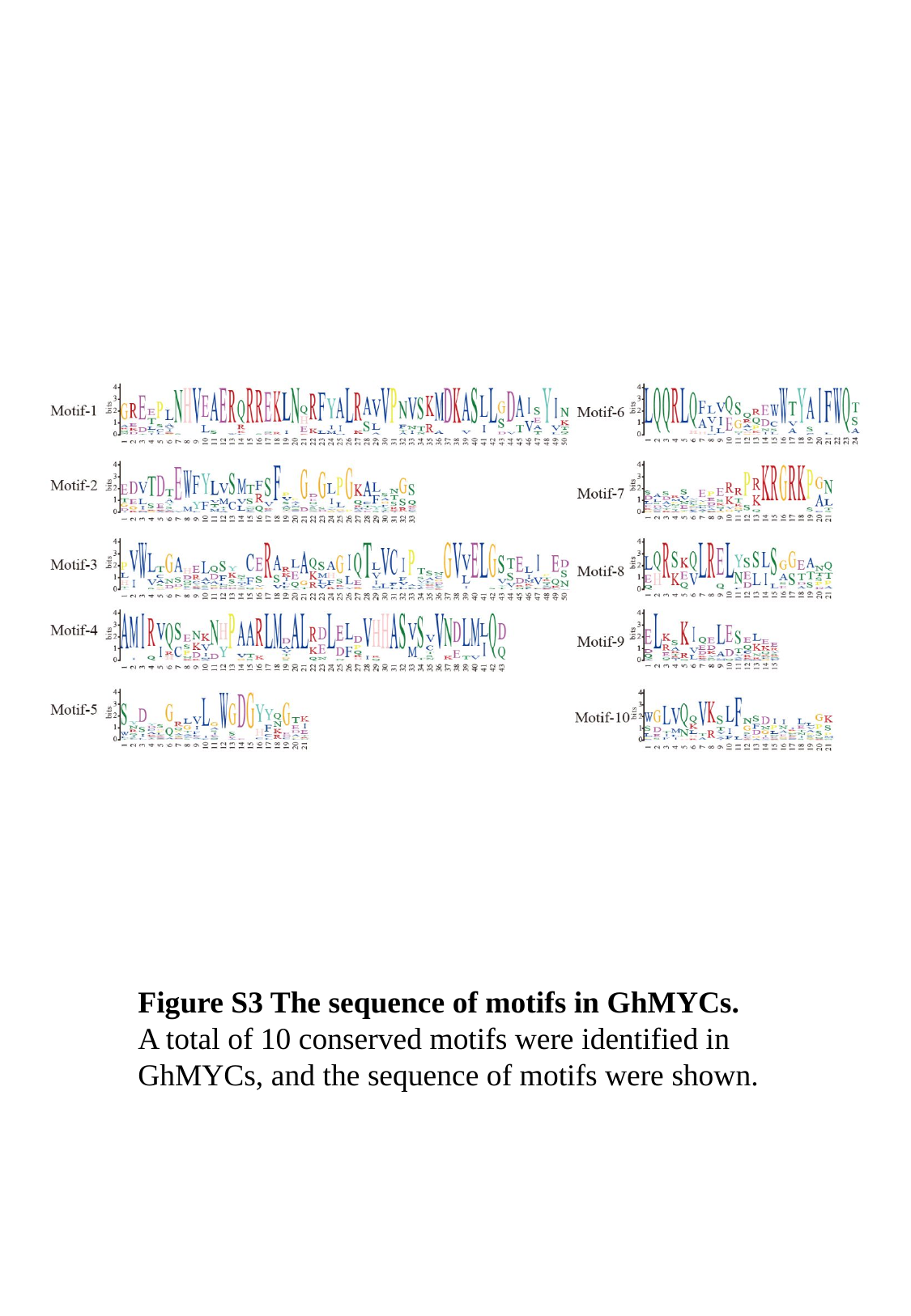

Figure S3 The sequence of motifs in GhMYCs.
A total of 10 conserved motifs were identified in GhMYCs, and the sequence of motifs were shown.

## Slide 4
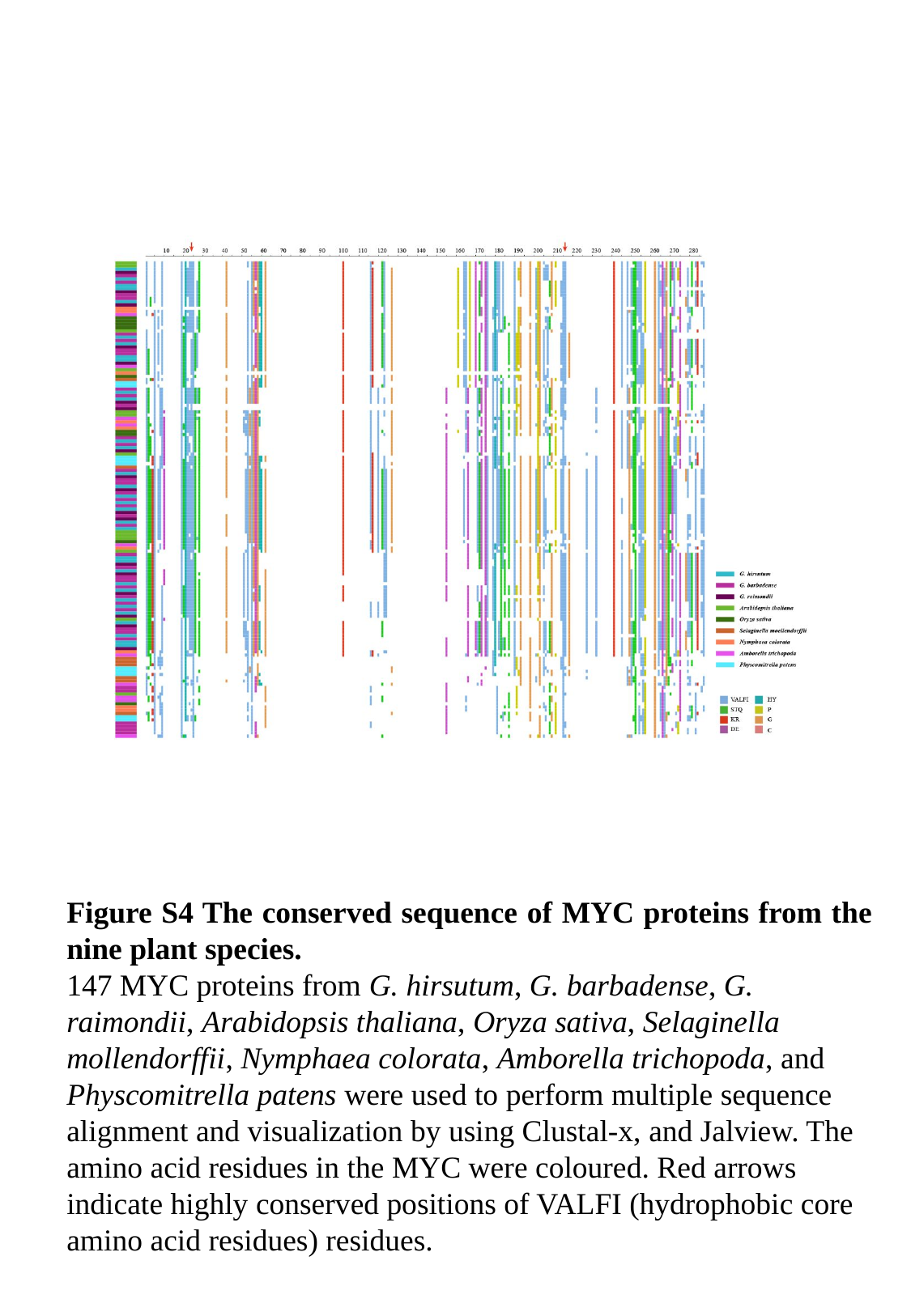

Figure S4 The conserved sequence of MYC proteins from the nine plant species.
147 MYC proteins from G. hirsutum, G. barbadense, G. raimondii, Arabidopsis thaliana, Oryza sativa, Selaginella mollendorffii, Nymphaea colorata, Amborella trichopoda, and Physcomitrella patens were used to perform multiple sequence alignment and visualization by using Clustal-x, and Jalview. The amino acid residues in the MYC were coloured. Red arrows indicate highly conserved positions of VALFI (hydrophobic core amino acid residues) residues.

## Slide 5
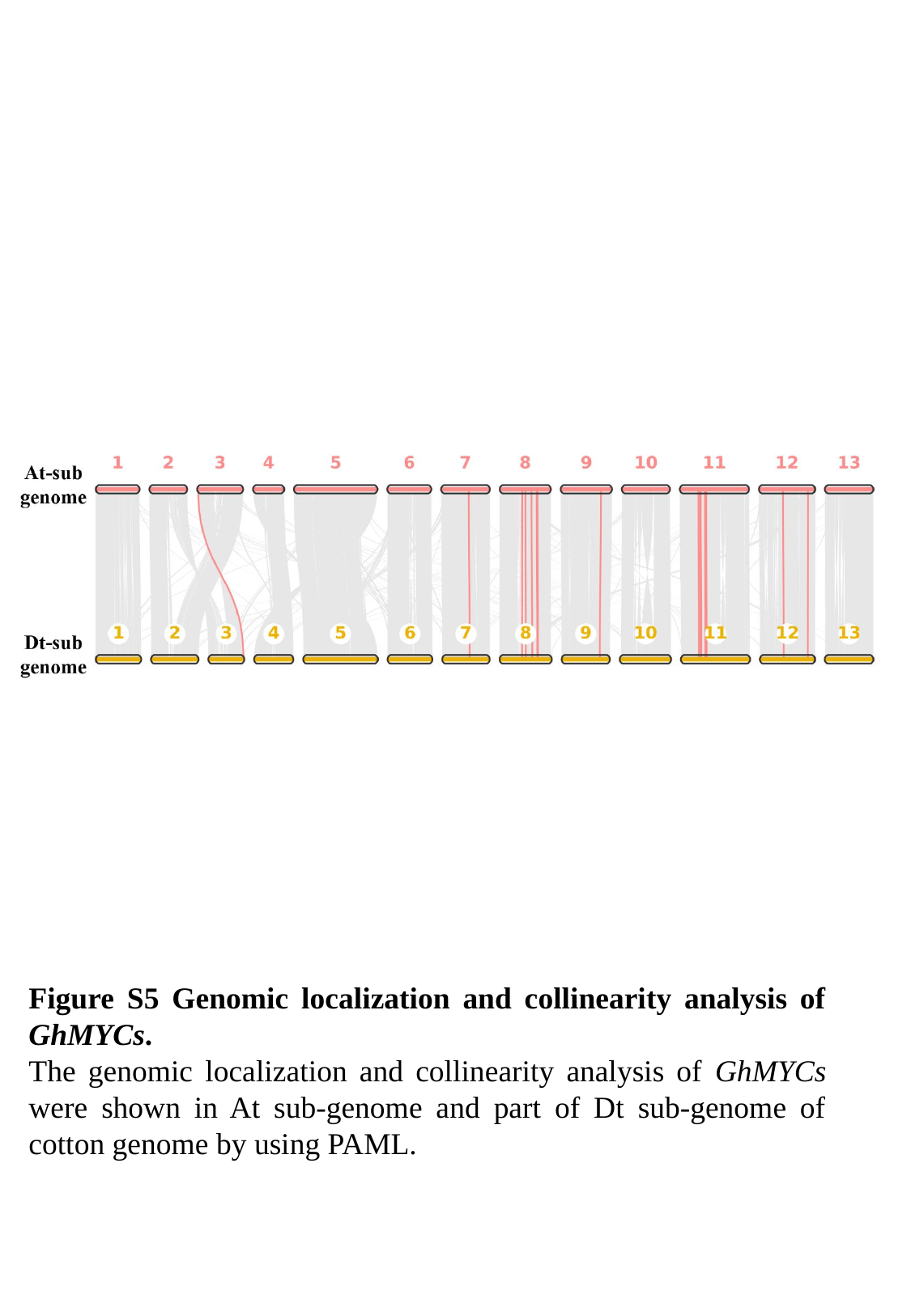

Figure S5 Genomic localization and collinearity analysis of GhMYCs.
The genomic localization and collinearity analysis of GhMYCs were shown in At sub-genome and part of Dt sub-genome of cotton genome by using PAML.
